# Tyrosinase-Deficient Skin Melanophore Lineage in *Xenopus tropicalis* Tadpoles Shows Strong Autofluorescence

**DOI:** 10.3390/ijms27031367

**Published:** 2026-01-29

**Authors:** Yuyan Jiang, Yijian Chen, Zeri Huang, Lian Chen, Xiao Huang

**Affiliations:** Institute of Cellular and Developmental Biology, College of Life Sciences, Zhejiang University, Hangzhou 310058, China; 12107043@zju.edu.cn (Y.J.); 12307085@zju.edu.cn (Y.C.); 3220102830@zju.edu.cn (Z.H.); chenlianzju@zuaa.zju.edu.cn (L.C.)

**Keywords:** *X. tropicalis*, melanophores, autofluorescence, gene editing, *Gch2*

## Abstract

Tyrosinase, encoded by *Tyr*, is a key rate-limiting enzyme in melanin biosynthesis. Knockout of *Tyr* results in a distinct albino phenotype, making it a widely used target for evaluating gene-editing efficiency. Here, we found that the tyrosinase-deficient skin melanophore lineage of *Xenopus tropicalis* (*X. tropicalis*) tadpoles shows strong autofluorescence under the GFP filter, which may interfere with in vivo fluorescence imaging. Through spectral scanning analysis, we characterized the emission spectrum of the autofluorescence under commonly used excitation wavelengths for fluorescent proteins. Based on this, we established a reference protocol for identifying and excluding such interference in *Tyr*-targeted knockin studies. Furthermore, knockout of the GTP cyclohydrolase 2 gene (*Gch2*) using CRISPR-Cas9 significantly reduced the fluorescence intensity induced by tyrosinase deficiency, indicating an essential role of the enzyme and its mediated pterine biosynthesis in the generation of the autofluorescence. This study systematically characterized these fluorescent mutant melanophores in *X. tropicalis* tadpoles, providing a practical basis for avoiding fluorescent interference in experimental science and a new perspective on pigment cell development and evolution.

## 1. Introduction

Animal skin is covered with diverse pigment cells, which collectively form species-specific body color patterns [1,2,3]. These patterns not only serve as visual recognition cues but also confer multiple survival advantages to animals, such as ultraviolet radiation resistance, predator avoidance, and mate attraction [1,2,3]. Unlike mammals and birds, which possess only a single type of pigment cell (melanocytes), teleosts have evolved a richer variety of pigment cell types, including melanophores, iridophores, xanthophores, and leucophores [4,5]. The chromatic diversity of these cells stems from their specialized subcellular pigment organelles: melanophores and xanthophores achieve their specific pigment deposition through light-absorbing organelles known as melanosomes and pterinosomes, respectively [6,7]; leucophores are characterized by leucosomes and appear white, whereas iridophores produce a metallic sheen through their reflecting platelets [4,8].

Except for retinal pigment epithelium, all pigment cells in vertebrates originate from the neural crest [9,10]. In amphibians, neural crest stem cell populations typically differentiate into three types of pigment cells: melanophores, iridophores, and xanthophores [11]. In certain teleosts, the pigment cell lineage has further evolved leucophores [5,8], even though these cells exhibit characteristics similar to xanthophores during fate determination and differentiation [12,13]. In recent years, two distinct leucophore subtypes have been identified in zebrafish (generally considered to lack typical leucophores), termed xantholeucophores and melanoleucophores [14], demonstrating the high complexity and plasticity of pigment cell lineage differentiation. Notably, leucophores in medaka and Arabian killifish emit bright green fluorescence under blue light excitation [4,13], the characteristic also exhibited by xanthophores in zebrafish [15]. Importantly, the synthesis of these fluorescent pigments, distributed in different pigment cell types across various species, all relies on the pterine biosynthesis mediated by GTP cyclohydrolase 2 [4,13,15,16]. In amphibians, autofluorescent cells were also observed in the periodic albino (ap/ap) mutant of *Xenopus laevis* (*X. laevis*), specifically within regions destined for melanophores [17,18], yet the molecular basis of this fluorescence remains elusive. Focusing on conserved pigment cell phenotypes and pigment synthesis pathways across species may provide critical clues for understanding the evolutionary mechanisms of pigment cells.

*X. tropicalis*, a diploid amphibian model organism, shares 79% homology with the human genome, making it widely used in developmental genetics and cancer biology [19,20]. For instance, the successful generation of colorless and immunodeficient *X. tropicalis* strains has provided an excellent in vivo imaging tool for investigating tumorigenesis and metastasis mechanisms following allotransplantation [21]. Malignant transformation of melanocytes is a key driver of melanoma, a fatal cancer whose pathological processes are closely linked to the aberrant reactivation of neural crest melanocyte development [22,23]. Due to its similar skin anatomy to humans and high gene-editing efficiency, various melanoma disease models have been established based on *X. tropicalis* [24,25,26]. Therefore, in-depth characterization of pigment cell biology in *X. tropicalis* provides critical insights into the pathogenic mechanisms underlying melanoma. Additionally, compared to mice and zebrafish, *X. tropicalis* possesses the unique advantage of high fecundity (up to 8000 eggs per spawning) [20], and a series of significant advances in the development and application of gene-editing technology has been achieved in this species [26,27,28]. Tyrosinase, encoded by the gene *Tyr*, is a core enzyme required for melanin biosynthesis. Studies have confirmed that knockout of this gene leads to an albino phenotype, characterized by abolished melanin synthesis in skin melanocytes and retinal pigment epithelial cells [29,30,31]. Given the easily observable phenotype, multiple gene knockin techniques developed in *X. tropicalis* have selected the *Tyr* locus as the preferred target, while concurrently introducing fluorescent protein reporter genes as a validation strategy [29,32,33].

In this study, we unexpectedly discovered that mutant melanophores (MMs) in the skin of *X. tropicalis* tadpoles exhibited strong fluorescence under the GFP filter following targeted knockout of *Tyr*. This finding implies potential interference from such autofluorescence in the development of gene knockin technologies based on the *Tyr* locus as well as the applications of colorless *Xenopus* models. Therefore, we systematically analyzed the physiological profiles and autofluorescence spectra of MMs induced by *Tyr* knockout and phenylthiourea (PTU, a tyrosinase inhibitor) treatment, providing an experimental basis for identifying and eliminating such fluorescence interference. Furthermore, we identified and functionally characterized the GTP cyclohydrolase II gene (*Gch2*) in *X. tropicalis*, and found that knockout of *Gch2* led to a severe loss of fluorescence in tyrosinase-deficient MMs. This study not only offers critical cautions and references for in vivo fluorescence imaging experiments based on tyrosinase-deficient *X. tropicalis* but also provides new insights into the developmental and evolutionary mechanisms of pigment cells.

## 2. Results

### 2.1. Tyrosinase-Deficient Skin MMs in X. tropicalis Tadpoles Show Strong Autofluorescence

**Figure 1:**
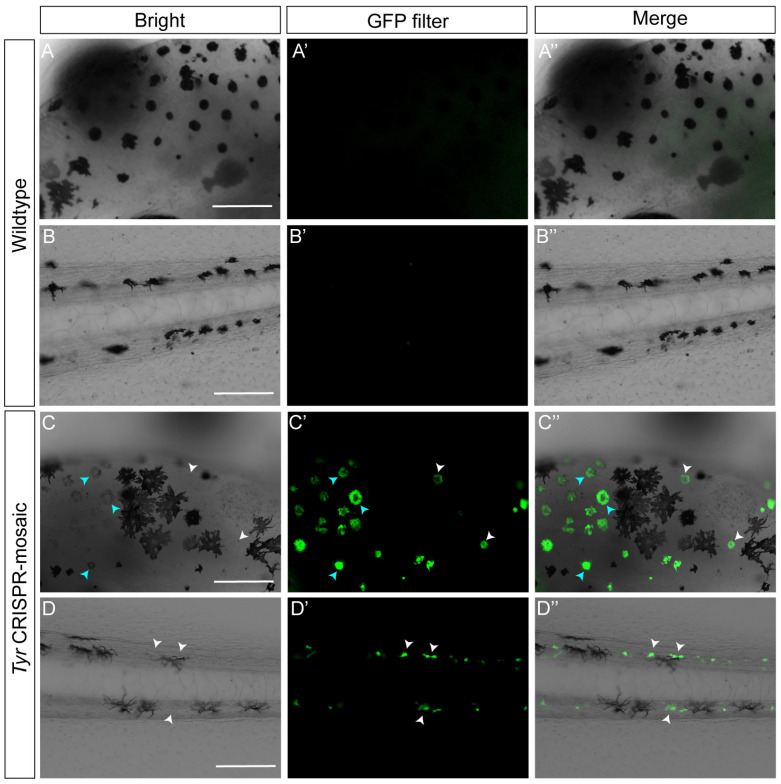
Knockout of *Tyr* induces strong fluorescence in skin MMs of *X. tropicalis* tadpoles under GFP filter. (**A**–**D**) Images of the dorsal head (**A**) and tail (**B**) skin of wild-type tadpoles (**A**), and the dorsal head (**C**) and tail (**D**) skin of F0 *Tyr*-knockout tadpoles under transmission light. (**A′**–**D′**) Corresponding fluorescence images under GFP filter. (**A″**–**D″**) Merged images of transmitted light and fluorescence channels. MMs that appear gray due to oocyte-derived melanin in the dorsal skin of F0 *Tyr*-knockout tadpoles (**C**–**C″**). The cyan and white arrowheads indicate gray and colorless MMs under transmitted light, respectively. Scale bars: 100 μm.

In recent years, the advancement of CRISPR technology has significantly expanded the genetic manipulation potential of *X. tropicalis*. When performing CRISPR-mediated knockout of *Tyr* in *X. tropicalis*, we unexpectedly observed a population of cells exhibiting strong fluorescence in the skin of stage 42 F0 tadpoles under the GFP filter (Figure 1C–C″,D–D″). Although xanthophores in the skin of *X. laevis* tadpoles appear to exhibit autofluorescence [17], it should be noted that xanthophores first emerge at stages 45/46 [34], suggesting that the fluorescence we observed is not derived from xanthophores. More importantly, there was no detectable fluorescence in the skin of wild-type tadpoles from the same batch under the GFP filter (Figure 1A–A″,B–B″). Notably, the redistribution of oocyte-derived melanin resulted in a portion of MMs in the dorsal head skin of the edited mosaic tadpoles appearing as discernible gray under transmitted light [21] (Figure 1C). Furthermore, we found that the positions of these gray MMs completely overlapped with the fluorescence signals under the GFP filter (Figure 1C–C″), demonstrating that this autofluorescence is derived from MMs induced by *Tyr* knockout.

**Figure 2:**
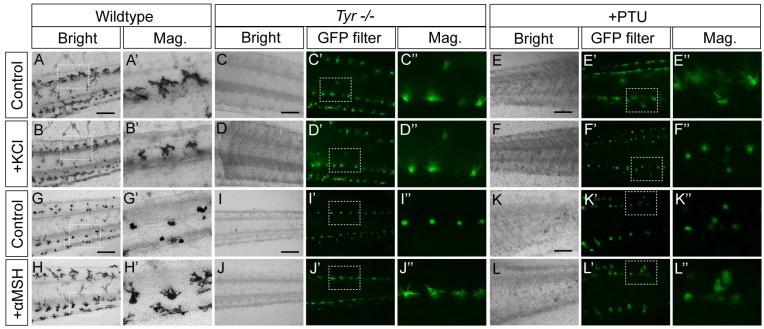
Physiological characterization of the skin Td-MMs in *X. tropicalis* tadpoles. (**A**–**L**) Transmission light images of excised tails from the three types of tadpoles before and after KCl treatment under physiological conditions (**A**–**F**), as well as before and after α-MSH treatment in BSS solution (**G**–**L**). Local magnified views of the transmission light images (**A′**,**B′**,**G′**,**H′**). Corresponding fluorescence images (**C′**–**F′**,**I′**–**L′**) and local magnified views (**C″**–**F″**,**I″**–**L″**) under GFP filter. Scale bar: 200 μm.

To rule out potential interference from genetic manipulation, we further performed imaging analysis on tails of stage 42 wild-type, *Tyr*-homozygous knockout (*Tyr*^−/−^) and PTU-treated tadpoles. Notably, melanophores are the only pigment cell type present in the tadpole tail at this developmental stage [17,18]. The results showed that melanophores in wild-type tadpole tails were scattered individually between glands (Figure 2A). In contrast, MMs in the corresponding regions of both *Tyr*^−/−^ and PTU-treated tadpole tails exhibited an albino phenotype under transmitted light (Figure 2C,E) and strong autofluorescence under the GFP filter (Figure 2C′,E′). These findings further indicate that tyrosinase-deficiency-induced MMs (Td-MMs) in the skin of *X. tropicalis* tadpoles possess strong autofluorescence.

### 2.2. Characterization of the Skin Td-MMs in X. tropicalis Tadpoles

To identify the autofluorescence of the two types of Td-MM, we characterized their physiological profiles by systematically assessing their dynamic responses to different chemical signals. Wild-type melanophores and both types of Td-MMs in the excised tails of stage 42 tadpoles exhibited dendritic morphology under physiological conditions (Figure 2A′,C″,E″), but contracted morphology in BSS saline solution [17] (Figure 2G′,I″,K″). Upon addition of KCl (150 mM) to explants under physiological conditions and α-MSH (0.6 µM) to those in BSS, wild-type melanophores displayed marked contraction (Figure 2A′,B′) and dispersion (Figure 2G′,H′) responses, respectively, which is consistent with previous characterizations of melanophores [4,17]. Similar to wild-type melanophores, MMs from PTU-treated tadpoles responded robustly to both chemical signals (Figure 2E″,F′’,K″,L″); in contrast, MMs from *Tyr^−/−^* tadpoles only exhibited a marked response to α-MSH (Figure 2C″,D″,I″,J″). These results can provide a basis for identifying the autofluorescence of these two types of Td-MMs in live cell fluorescence imaging.

**Figure 3:**
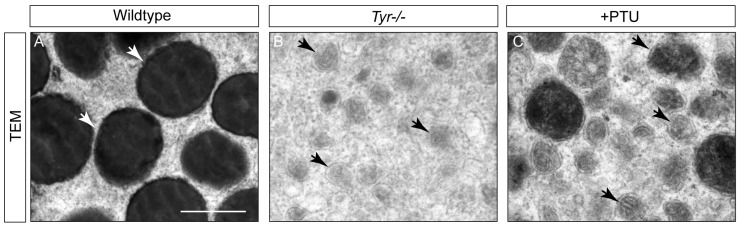
Ultrastructural analysis of the skin Td-MMs in *X. tropicalis* tadpoles. (**A**–**C**) Transmission electron microscopy (TEM) images showing the ultrastructure of skin melanophores in wild-type tadpoles (**A**), and MMs in *Tyr^−/−^* (**B**) and PTU-treated (**C**) tadpoles. White and black arrowheads indicate mature and immature melanosomes, respectively. Due to the presence of melanin derived from oocytes, the melanosomes in the MMs of PTU-treated tadpoles show a higher degree of maturation compared to those in *Tyr^−/−^* tadpoles. Scale bar: 0.5 μm.

In melanophores, endosome-derived vacuoles develop into mature melanosomes through the formation of internal striations and deposition of melanin within them [6]. Previous studies have reported a type of special pigment cell with autofluorescence in the skin of ap/ap mutant *X. laevis* [17]. As their organelles contain reflecting platelets derived from immature melanosomes and appear white under reflected light, these cells were named “white pigment cells” [17,18]. However, the skin Td-MMs in *X. tropicalis* tadpoles do not appear white under reflected light [32], and thus we speculate that these two cell types are distinct in identity. To test this hypothesis, we performed ultrastructural analysis of the skin MMs in *Tyr*^−/−^ and PTU-treated tadpoles using transmission electron microscopy (TEM). Our results showed that both types of Td-MMs exclusively possessed abundant immature melanosomes, without any reflecting platelet structures detected (Figure 3A–C). This result confirms that the skin Td-MMs are not the same cell types as the “white pigment cells” found in ap/ap mutant *X. laevis*.

### 2.3. Spectroscopic Analysis of Autofluorescence in Td-MMs

**Figure 4:**
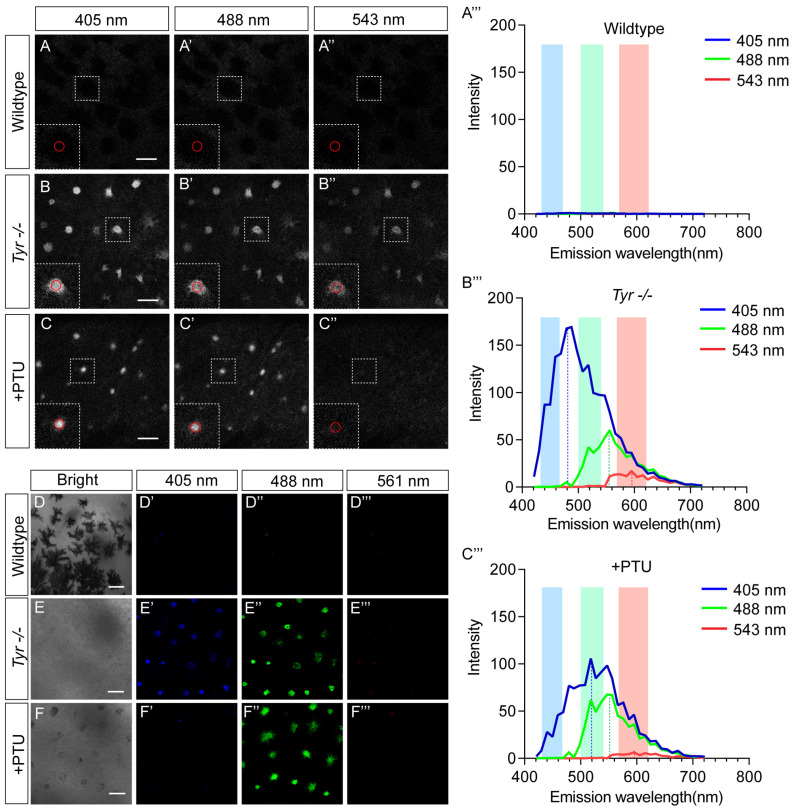
Spectral analysis of autofluorescence and assessment of fluorescence interference from Td-MMs. Images showing full-spectrum emission signals of skin melanophores in the wild-type tadpoles (**A**–**A″**), and skin MMs in the *Tyr^−/−^* (**B**–**B″**) and the PTU-treated (**C**–**C″**) tadpoles under excitation at different wavelengths (405, 488, 543 nm). (**A‴**–**C‴**) Graphs showing the normalized emission spectra corresponding to the red-circled regions, and the emission peaks under each excitation wavelength are indicated by dashed lines. The conventional emission bands of each fluorescent protein are shown with different colored boxes (TagBFP: 430–470 nm, GFP: 500–540 nm, mCherry: 570–620 nm). Images of these tadpole skin under transmission light (**D**–**F**) and fluorescence images acquired within the conventional emission bands of TagBFP (**D′**–**F′**), EGFP (**D″**–**F″**), and mCherry (**D‴**–**F‴**) under the three excitation wavelengths. Scale bars: 50 μm.

The differences in physiological characteristics (responsiveness to KCl) between skin MMs in *Tyr^−/−^* and PTU-treated tadpoles suggested a potential distinction in their spectral characteristics. Meanwhile, the autofluorescence could interfere with in vivo fluorescence imaging assays. To address these points, we used λ spectral scanning technology to analyze the autofluorescence spectral characteristics in wild-type melanophores (as a control) and the two types of Td-MMs. Three common excitation wavelengths (405 nm, near-UV; 488 nm, blue; 543 nm, green) were used, and emission spectra were collected, respectively. The results showed that wild-type melanophores exhibited no detectable fluorescence emission under all three excitation conditions, consistent with the broad-spectrum absorption properties of melanin [1] (Figure 4A–A‴). In contrast, both types of Td-MMs exhibited robust emission signals upon excitation at 405 nm and 488 nm (Figure 4B–B‴,C–C‴). Notably, while both types of Td-MMs emitted a broad spectrum spanning 400–700 nm under 405 nm excitation, their spectral features differed markedly: MMs induced by PTU treatment had an emission peak at ~480 nm, whereas the emission peak in *Tyr*-knockout-induced MMs showed a redshift to ~520 nm (Figure 4B‴,C‴). Furthermore, the two types of Td-MMs shared similar emission spectral features under 488 and 543 nm excitation. Specifically, excitation at 488 nm elicited emission initiation at ~490 nm with a peak at ~550 nm, while excitation at 543 nm led to emission initiation at ~550 nm with a peak at ~600 nm (Figure 4B‴,C‴). It should be noted that compared with 405 and 488 nm excitation, both types of Td-MMs showed the narrowest emission spectra and weakest intensity under 543 nm excitation (Figure 4B–B‴,C–C‴). This trend suggests that green light and longer-wavelength excitation cannot effectively trigger their fluorescence. Collectively, these results imply subtle variations in fluorescent pigment composition between these two types of Td-MMs and, more importantly, provide key data to enable the elimination of such autofluorescence interference in imaging applications.

Blue fluorescent protein TagBFP, green fluorescent protein GFP, and red fluorescent protein mCherry are commonly used representatives in in vivo fluorescence imaging. Their fluorescence signals are typically acquired under excitation at 405 nm, 488 nm, and 561 nm, respectively [35]. To assess whether the autofluorescence interferes with the signal detection of these fluorescent proteins, we collected fluorescence signals from wild-type melanophores and two types of Td-MMs within the conventional emission bands of each fluorescent protein [35]. As expected, wild-type melanophores showed no clear fluorescence under all three excitation wavelengths (Figure 4D–D‴), nor did either type of Td-MM under 561 nm excitation (Figure 4E‴,F‴). Additionally, both types of Td-MMs exhibited strong fluorescence under 488 nm excitation (Figure 4E″,F″), consistent with their emission peaks being close to the EGFP acquisition band (500–540 nm, the green box) (Figure 4B‴,C‴). Notably, only MMs in *Tyr^−/−^* tadpoles showed significant fluorescence under 405 nm excitation among the two types of Td-MMs (Figure 4E′); this was due to the TagBFP acquisition band (430–470 nm, the blue box) being adjacent to the emission peak of this cell type (Figure 4B‴), but only covering a small initial part of the emission spectra of MMs induced by PTU treatment (Figure 4C‴). Taken together, these results suggest that employing far-red fluorescent proteins such as mCherry can effectively eliminate autofluorescence interference for in vivo imaging of tyrosinase-deficient *X. tropicalis* tadpoles.

### 2.4. In Vivo Fluorescence Imaging in X. tropicalis Models with Tyr-Targeted Gene Knockin

**Figure 5:**
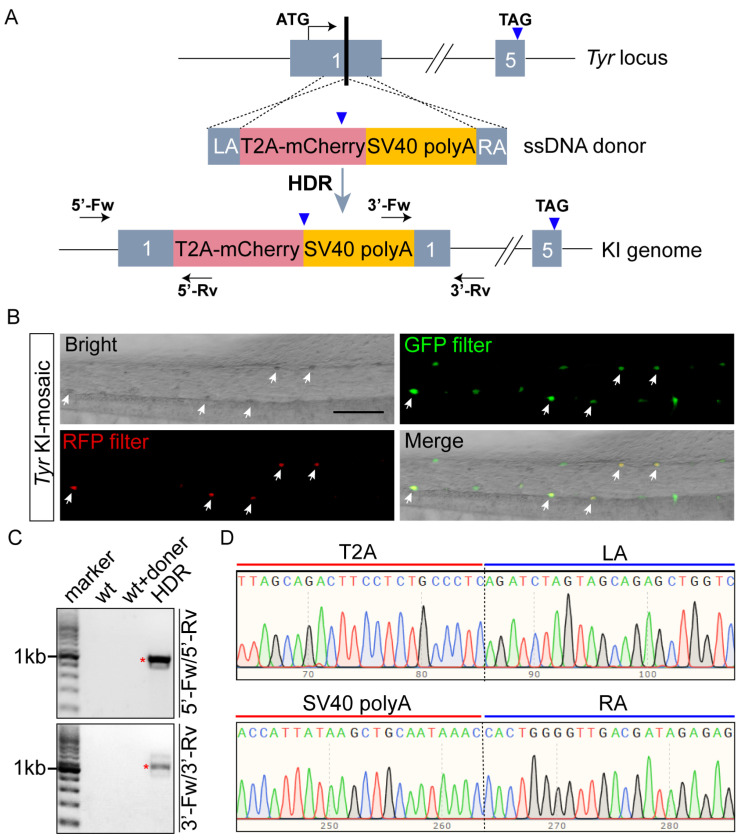
HDR-mediated seamless integration of mCherry into the *Tyr* locus of *X. tropicalis*. (**A**) Schematic diagram showing the gene-editing strategy. The ssDNA donor contains a left homologous arm (LA) and right homologous arm (RA) of 100 nt each, flanking the T2A protease cleavage sequence followed with the mCherry coding region, and the SV40 polyA signal. Upon HDR occurrence, the T2A-mCherry-SV40 polyA cassette is inserted in-frame into the middle of exon 1, leading to the expression of a fusion protein consisting of the truncated Tyr mutant and T2A-mCherry. mCherry is released via proteolytic cleavage in the *Tyr*-expressing cell lineage, resulting in loss-of-function of Tyr with concurrent red fluorescence in MMs. Positions of PCR primers for F0 genotyping are indicated in the genome DNA (**A**). Stop codons are marked with blue inverted triangles. (**B**) Imaging of F0 tadpole tail under transmission light, GFP, and RFP filter. MMs exhibited two fluorescence profiles: (i) concurrent red and green fluorescence (mCherry+/GFP+, white arrowheads), indicating successful HDR; (ii) green fluorescence only (mCherry-/GFP+), indicating no HDR but frameshift mutations. Scale bar: 100 μm. (**C**) Agarose gel electrophoresis images showing the results of F0 genotyping PCR using 5′ junction primers (5′-Fw/5′-Rv) and 3′ junction primers (3′-Fw/3′-Rv). Results showed that the two expected bands (marked by red asterisks) were both amplified exclusively from the HDR-positive (mCherry+) tadpole genome, while no corresponding bands were detected in either wild-type tadpoles or those injected with donor DNA only. (**D**) TA cloning and sequence mapping of the expected bands (from (**C**)) confirmed seamless integration of the knockin fragment at the target locus.

*Tyr* is one of the commonly used targets in the development of gene knockin techniques. By introducing a fluorescent reporter gene into its coding region, the occurrence of knockin events can be determined via fluorescence signals in MMs [28,32,36]. To validate the reliability of mCherry as a reporter in the *Tyr*-targeted knockin system based on *X. tropicalis*, we established a corresponding gene knockin model. In this model, the CRISPR/Cas9 system was utilized to induce double-strand breaks (DSBs) in the coding region of the first exon of *Tyr*; a donor template flanked by homology arms was then integrated into the target site via homology-directed repair (HDR), allowing the endogenous *Tyr* promoter to drive a truncation mutant and T2A-mCherry (Figure 5A). Due to the mosaic effect of gene knockin, four potential gene-editing outcomes may exist in the *Tyr*-expressing cell lineage of F0 tadpoles: First, biallelic HDR occurs simultaneously (with a low frequency). Second, HDR occurs in one allele while frameshift mutations occur in the other. Both scenarios lead to loss-of-functions of *Tyr*, resulting in an albino phenotype and concurrent expression of *mCherry* (with distinct red fluorescence). Third, HDR occurs in one allele with no editing in the other. Although *mCherry* is still expressed under this condition, fluorescence is hardly detectable due to the presence of melanin. Fourth, neither allele undergoes HDR but frameshift mutations occur, leading to an albino phenotype without red fluorescence expression.

Subsequently, we injected the Cas9 protein-sgRNA complex and the single-stranded DNA (ssDNA) donor into the embryo, and we imaged F0 tadpoles to detect gene knockin events. Our result showed that cells with green fluorescence under the GFP filter (GFP+) uniformly showed an albino phenotype under transmission light, whereas only a fraction of these cells exhibited red fluorescence under the RFP filter (mCherry+) (Figure 5B). This result indicates that at least one allele underwent HDR in “GFP+/mCherry+” cells, while no HDR occurred in either allele of “GFP+/mCherry-” cells, with only frameshift mutations present. Further genotyping results revealed that the expected-size bands corresponding to the 5′-junction and 3′-junction were amplified specifically from F0 tadpoles with HDR (mCherry+), with no such bands detected in wild-type or donor-only injected controls (Figure 5C). TA cloning coupled with sequence mapping confirmed precise integration of the knockin cassette at the target locus (Figure 5D), validating the reliability of the mCherry reporter signal. Collectively, these findings establish mCherry as a reliable fluorescent reporter for in vivo imaging applications in tyrosinase-deficient *X. tropicalis*.

### 2.5. The Fluorescence Intensity of Skin MMs in Tyr^−/−^ X. tropicalis Tadpoles Is Higher than That of Xanthophores in Zebrafish

**Figure 6:**
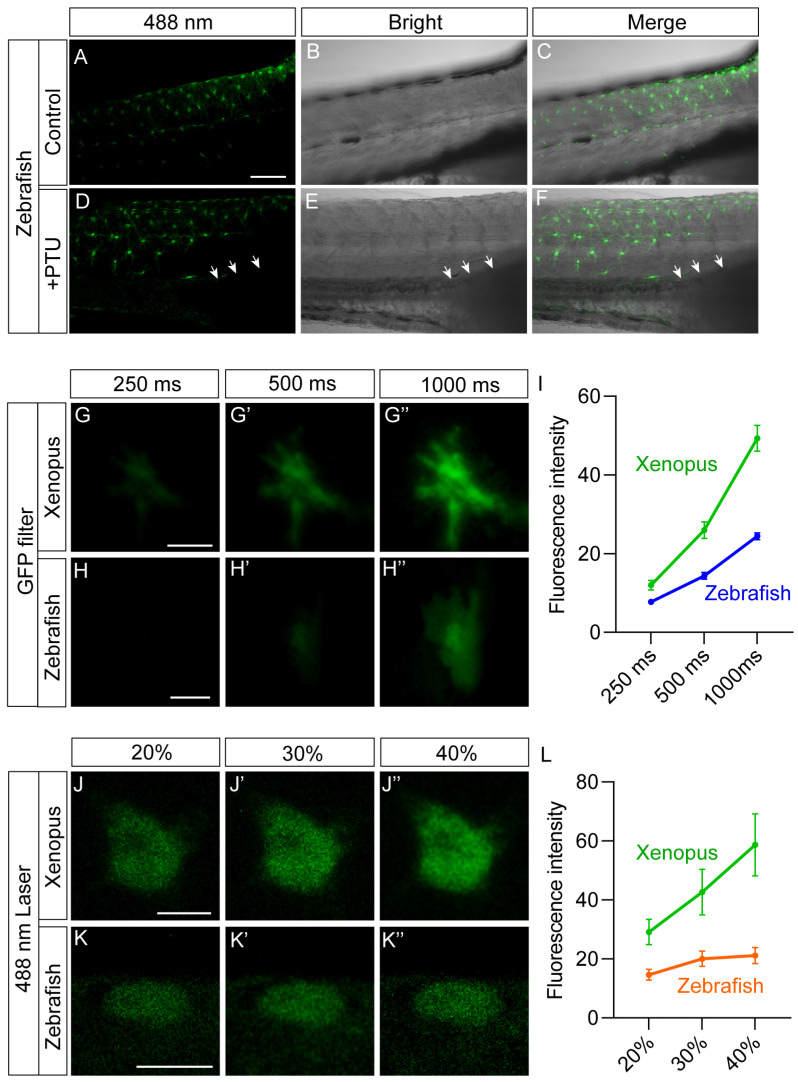
Skin MMs in *Tyr^−/−^* tadpoles are brighter than xanthophores in zebrafish. (**A**–**F**) Fluorescence, transmission light, and merged images of the trunk region in wild-type (**A**–**C**) and PTU-treated (**D**–**F**) 5 dpf zebrafish under 488 nm excitation (with 500–540 nm emission). Regions destined to form melanophores exhibit an albino phenotype (white arrowheads), but no fluorescence in the PTU-treated fish. Scale bar: 100 μm. Fluorescence images of skin MMs in *Tyr^−/−^* tadpoles (**G**–**G″**) and xanthophores in 5 dpf zebrafish (**H**–**H″**) at different exposure times under LED excitation, with quantitative analysis (**I**). Scale bar: 20 μm. Fluorescence images of MMs in *Tyr^−/−^* tadpoles (**J**–**J″**) and xanthophores in 5 dpf zebrafish (**K**–**K″**) under 488 nm excitation at varying laser powers, with quantitative analysis (**L**). Scale bar: 10 μm. Number of cells analyzed, LED excitation: MMs, n = 13; xanthophores, n = 12; 488 nm laser excitation: MMs, n = 5; xanthophores, n = 7. Data are presented as mean ± SEM.

Previous studies have reported that xanthophores in zebrafish emit bright green fluorescence under blue light excitation [15]. We imaged wild-type zebrafish at 5 days post-fertilization (dpf5) using 488 nm excitation (with 500–540 nm emission), confirming that there were abundant autofluorescent cells in the trunk region (Figure 6A–C). To further investigate whether the tyrosinase-deficient melanophore lineage in zebrafish can also exhibit autofluorescence similar to those in *X. tropicalis*, we treated sibling zebrafish embryos with PTU and imaged the dpf5 embryo. The results showed that PTU treatment led to an albino phenotype in regions normally occupied by melanophores (Figure 6B,E). However, these regions did not show any autofluorescence (Figure 6D–F), indicating that the autofluorescence characteristic of Td-MMs is not conserved among species.

Notably, the parameter heterogeneity of various fluorescence imaging equipment limits the standardized quantification of the fluorescence intensity of Td-MMs in *X. tropicalis*. To address this, we performed a comparative analysis using xanthophores in zebrafish as a reference. Fluorescence imaging of both skin MMs in *Tyr^−/−^ X. tropicalis* tadpoles and xanthophores in dpf5 zebrafish was performed using both an LED fluorescence microscope (with GFP filter) and a laser scanning confocal microscope, followed by intensity quantification with ImageJ 1.8.0 software to enable a quantitative comparison. The results showed that under LED light excitation, the fluorescence intensity of skin MMs in *Tyr^−/−^* tadpoles was consistently higher at different acquisition times (250, 500, and 1000 ms) (Figure 6G,G′,H,H′), approximately twice that of xanthophores in zebrafish (Figure 6I). Additionally, this two-fold difference was maintained even under low-power 488 nm excitation (10% and 20% laser power) (Figure 6J,J′,K,K′,L). However, at 30% laser power, the fluorescence intensity of xanthophores in zebrafish reached saturation (Figure 6K′,K″,L), whereas that of MMs in *Tyr^−/−^* tadpoles continued to increase (Figure 6J′,J″,L). Taken together, these results indicate that the fluorescence intensity of MMs in *Tyr^−/−^* tadpoles is significantly higher than that of xanthophores in zebrafish.

### 2.6. Gch2 Is Essential for Fluorescent Pigment Synthesis in Skin Td-MMs of X. tropicalis Tadpoles

**Figure 7:**
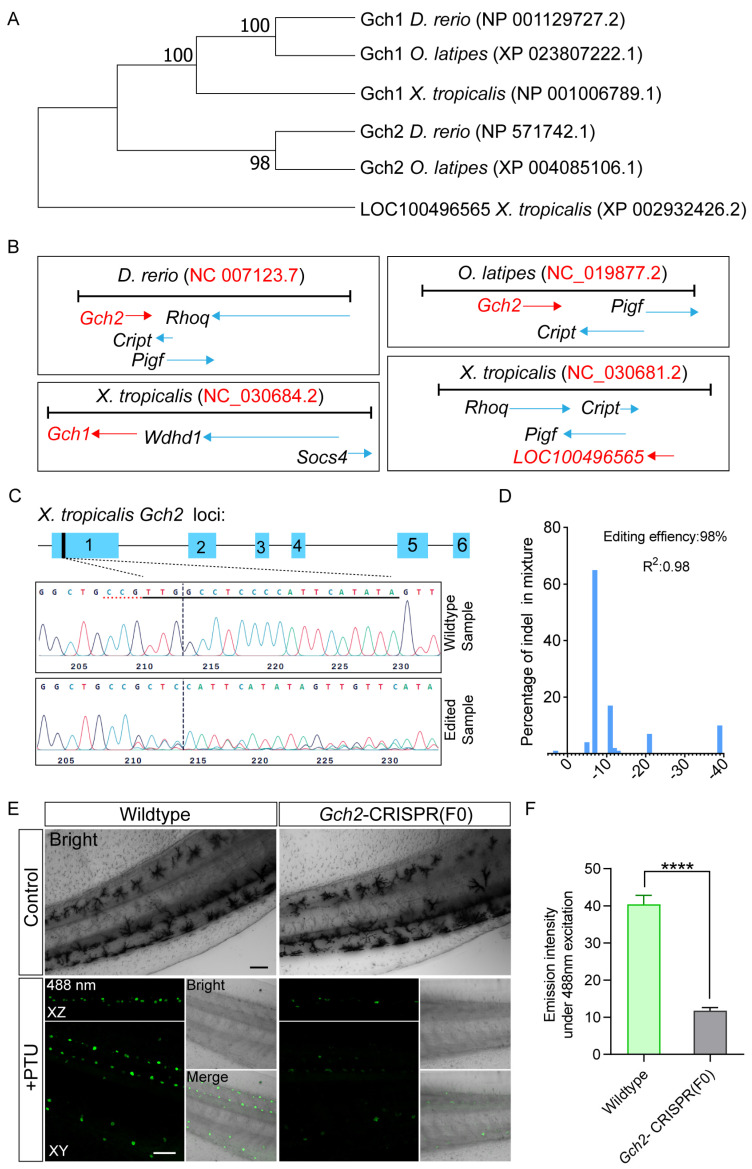
Knockout of *Gch2* leads to a severe loss of autofluorescence in skin Td-MMs of *X. tropicalis* tadpoles. (**A**) Phylogenetic tree constructed based on the amino acid sequences of zebrafish and medaka Gch1/Gch2, as well as *X. tropicalis* Gch1 and LOC100496565. The tree was built using the neighbor joining method and visualized with MEGA11 software. Node values indicate the percentage of replicate trees in which the associated branch clustered together in a bootstrap test with 1000 replicates. (**B**) Genomic loci of zebrafish and medaka *Gch2*, and of *X. tropicalis Gch1* and *LOC100496565* (red arrows), along with their adjacent loci (blue arrows). (**C**) The upper schematic showing gene structure of *X. tropicalis Gch2*, with the target site (black thick line) and sgRNA sequence (dashed line) indicated. The lower panel showing Sanger sequencing electropherograms of genomic fragments spanning the target site in wild-type and edited samples. The sgRNA target sequence (black underline) and PAM sequence (red dashed underline) in wild-type samples are marked, while the expected cleavage site is indicated by vertical dashed lines. (**D**) Analysis of indel sizes (x-axis) and their corresponding proportions (y-axis) calculated using the ICE tool. (**E**) images of stage 42 *Gch2*-knockout and sibling wild-type tadpoles, either PTU-treated or untreated, acquired under transmission light and 488 nm excitation (with 500–540 nm emission). For the 488 nm channel, fluorescence signals from z stack images are displayed in both XZ (side view) and XY (top view) planes. Scale bars: 100 μm. (**F**) Quantitative analysis of the fluorescence intensity of skin MMs from wild-type and *Gch2*-knockout tadpoles. Number of cells analyzed: wild type, n = 104; Gch2 knockout, n = 85. Data are presented as mean ± SEM; **** *p* < 0.0001.

Previous studies have reported that knockout of *Gch2* (NM_131667.1) in zebrafish led to abolishment of pterinosome-derived fluorescent pigmentation in xanthophores [15,16]. Although the autofluorescence of xanthophores is relatively weak (Figure 6I,L), we hypothesized that Gch2 might also play a role in fluorescent pigment synthesis in skin Td-MMs of *X. tropicalis* tadpoles. To identify the ortholog of *Gch2* in *X. tropicalis*, we performed a BLAST 2.17.0 alignment using the amino acid sequence of zebrafish Gch2 against the *X. tropicalis* protein database and identified two candidate proteins: Gch1 (NP_001006789.1) and LOC100496565 (XP_002932426.2) (Figure 7A). Notably, both genomes of zebrafish and medaka possess two paralogous genes, *Gch1* and *Gch2*. Medaka *Gch2* (XM_004085058.3) has been characterized as an early lineage marker for xanthophores and leucophores, and its encoded GTP cyclohydrolase 2 serves as a key enzyme for pterine biosynthesis [13]. To determine the evolutionary origin of these two candidate proteins, we first constructed an amino acid phylogenetic tree encompassing zebrafish and medaka Gch1/Gch2 as well as the candidates. We found that *X. tropicalis* Gch1 clustered with Gch1 orthologs from zebrafish and medaka (Figure 7A). Furthermore, genomic synteny analysis revealed that the *X. tropicalis* LOC100496565 locus shares completely conserved adjacent genes (*Rhoq*, *Cript*, *Pigf*) with the zebrafish *Gch2* locus (Figure 7B), and two conserved adjacent genes (*Cript*, *Pigf*) with the medaka *Gch2* locus (Figure 7B). These results strongly support that *X. tropicalis LOC100496565* is the ortholog of *Gch2*. Accordingly, we annotated *LOC100496565* as *Gch2*.

Subsequently, to verify the requirement of Gch2 for fluorescent pigment synthesis in skin Td MMs of *X*. *tropicalis* tadpoles, we performed knockout of *Gch2* by injecting Cas9 protein along with sgRNA into embryos. Cloning and sequencing of genomic fragments revealed extensive insertions/deletions (indels, up to 98%) near the target site in F0 edited samples (Figure 7C,D), indicating highly efficient knockout of *Gch2*. Then, we subjected a subset of F0 *Gch2*-knockout embryos and their wild-type siblings to parallel PTU treatment, and performed imaging of the stage 42 tadpoles under both transmission light and 488 nm laser excitation. As expected, both wild-type and *Gch2*-knockout tadpole skin in the PTU-treated group exhibited a complete albino phenotype (Figure 7E). However, in contrast to the strong fluorescence observed in wild-type tadpoles, the skin Td-MMs in *Gch2*-knockout tadpoles showed a severe reduction in fluorescence intensity (Figure 7E,F). Notably, both untreated wild-type and *Gch2*-knockout tadpole skin developed abundant melanophores (Figure 7E), indicating that knockout of *Gch2* does not affect melanophore formation. Collectively, these results demonstrate that Gch2 plays an essential role in fluorescent pigment synthesis in skin Td-MMs of *X. tropicalis* tadpoles.

## 3. Discussion

### 3.1. Origin of the Autofluorescence in Skin Td-MMs of X. tropicalis Tadpoles

Our study revealed that the skin melanophore lineage in tyrosinase-deficient *X. tropicalis* tadpoles shows strong fluorescence. Concurrently, this autofluorescent characteristic of MMs has only been reported in ap/ap mutant *X. laevis* [17], with no reports in other species. Consistent with previous reports that Td-MMs in Arabian killifish do not show fluorescence [4], we also failed to detect any fluorescence signals in Td-MMs of zebrafish (Figure 6D–F). Given that the melanin synthesis pathway is highly conserved across species [3,6], we hypothesize that the autofluorescence does not originate from metabolic byproducts resulting from blocked melanin synthesis.

Notably, in ap/ap mutant *X. laevis*, caused by structural deletion of the *Hps4* gene [37], two distinct populations of autofluorescent MMs are observed in the tadpole skin: one appears white under reflected light, with organelles containing reflecting platelets derived from immature melanosomes [17,38]; the other lacks white reflection, and its organelles only accumulate mature and immature melanosomes [17,18]. Our results demonstrated that immature melanosomes in skin Td-MMs of *X. tropicalis* tadpoles do not develop any reflecting platelets, suggesting that this autofluorescence is not derived from such structures. In Arabian killifish, knockout of *Gch* leads to a loss of autofluorescence in leucophores while preserving their white reflection [4], which supports that fluorescent pigments and white reflecting pigments are different molecules. On the other hand, the occurrence of autofluorescence in skin MMs induced by distinct genetic backgrounds (disruption of *Tyr*/*Hps4*) further suggests that the autofluorescence may originate from immature melanosomes.

### 3.2. Interferences and Applications of the Autofluorescence

For in vivo fluorescence imaging, endogenous fluorescence of experimental materials often interferes with signal detection and high-quality image acquisition [35]. Based on the full-spectrum emission data from this study, selecting appropriate fluorescent proteins and adjusting the emission band can effectively eliminate interference from autofluorescence of Td-MMs. While *X*. *tropicalis* serves as a classic model for early embryonic development [20], the lack of commercially available antibodies significantly limits molecular mechanism analysis and related research progress based on this model [27,29]. With the advancement of CRISPR/Cas9 technology, an increasing number of researchers have developed knockin techniques in *Xenopus*, providing a crucial tool for the precise fusion of fluorescent proteins or tags at endogenous gene loci [27,29,32,33]. Notably, for the development of gene knockin technology targeting the *Tyr* locus, the autofluorescence of MMs may interfere with GFP signal detection; however, using mCherry as the fluorescent reporter not only effectively avoids such interference but also enables precise tracing of MMs (Figure 5), which are not easily discernable in the brightfield. Based on this, the proportions of different editing events can be assessed by quantifying the numbers of “mCherry+/GFP+” and “mCherry-/GFP+” cells. It should be emphasized that in many *Tyr*-targeted gene-editing studies in *X. tropicalis*, signals from GFP reporters are not necessarily false positives, as genotyping is still the gold standard to verify successful gene editing.

### 3.3. Fluorescent Pigment Synthesis and Pigment Cell Evolution

**Figure 8:**
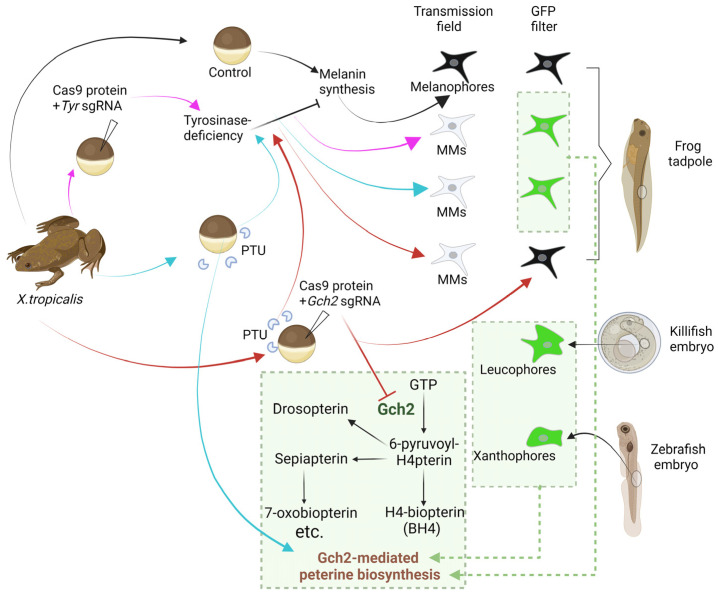
Summary of this study. In *X. tropicalis*, genetic (*Tyr* knockout) and chemical (PTU treatment) induction of tyrosinase deficiency both block the melanin synthesis and produce mutant melanophores (MMs) exhibiting the albino phenotype. Both types of MMs show strong autofluorescence under GFP filter. Critically, the leucophores of killifish and the xanthophores of zebrafish also show autofluorescence under the GFP filter, and their fluorescent pigment synthesis relies on the Gch2-mediated pterine biosynthesis. Furthermore, knockout of *Gch2* in *X. tropicalis* abolished the autofluorescence of tyrosinase-deficiency-induced MMs, demonstrating that the synthesis of fluorescent pigments in these distinct pigment cell types shares a conventional pathway. The pterine synthesis pathway is constructed by three major processes: H4-biopterin (BH4) is synthesized de novo from GTP (Guanosine Triphosphate) under the catalysis of Gch2. Concurrently, the intermediate product 6-pyruvoyl-H4pterin is converted into Drosopterin and Sepiapterin. Furthermore, Sepiapterine can be transformed into 7-oxobiopterin and other pterine compounds.

Our study demonstrates that fluorescent pigment synthesis in skin Td-MMs of *X. tropicalis* tadpoles relies on the Gch2-mediated pterine biosynthesis (Figure 7). This metabolic pathway has also been reported to play a key role in autofluorescence generation in leucophores of Arabian killifish [4] and xanthophores of zebrafish [16]. Unlike Td-MMs, however, leucophores in killifish exhibit strong fluorescence under both GFP and the RFP filter [4]. In addition, our results also show that the fluorescence intensity of MMs in *Tyr^−/−^* tadpoles is significantly higher than that of xanthophores in zebrafish. These facts suggest that the composition of fluorescent pigment, specifically the types and quantities of pterine compounds, likely varies among these species. Notably, previous studies have shown that Gch2 catalysis is required for the biosynthesis of multiple types of pterine compounds [39,40] (Figure 8); however, the specific pterine types that exhibit autofluorescent properties are not yet fully understood.

Two hypotheses may explain the mechanism underlying fluorescent pigment synthesis in skin Td-MMs of *X. tropicalis* tadpoles. First, it is possible that fluorescent pigment inherently exists in wild-type melanophores of *X. tropicalis*, where their autofluorescence is masked by melanin under physiological conditions and only becomes detectable when melanin synthesis is blocked. It has been suggested that the differentiation of melanophores into melanoleucophores in zebrafish could be an evolutionary outcome, which is achieved by co-option of purine biosynthetic and biomineralization machinery similar to those of iridophores to acquire guanine crystals [14]. Since autofluorescent Td-MMs have not been reported in other species, the recruitment of the pteridine biosynthesis pathway (typically restricted to xanthophores and leucophores) into *Xenopus* melanophores may suggest a case of adaptive evolution. It is possible that the *X. tropicalis* may have developed stronger reflecting pigment to resist the strong light in its tropical African habitats, even when melanin synthesis is impaired. The alternative hypothesis is that tyrosinase deficiency aberrantly activates the pterine biosynthesis within the melanophore lineage. Studies have reported that pterinosomes and melanosomes are homologous organelles, both originating from the Golgi-endoplasmic reticulum-lysosome (GERL) system [41]. As far back as 70 years ago, studies demonstrated that xanthophores can transform into melanophores in the context of interspecies transplantation [14,41]. These facts suggest that the pigment synthesis pathways in these two types of organelles may exhibit high plasticity under specific conditions. Regardless of which hypothesis holds true, our findings provide novel insights into the developmental and evolutionary mechanisms of pigment cells.

## 4. Materials and Methods

### 4.1. Plasmid Construction and ssDNA Synthesis

To construct the template plasmid for synthesizing the ssDNA donor in gene knockin experiments, the pMD18-T plasmid was first double-digested with *Sal* I and *Kpn* I restriction enzymes as the backbone. Then, the CDS fragments of Tyr were cloned as the left (100 bp: from 108 to 207) and right (100 bp: from 225 to 324) homologous arms, and T2A-mCherry and SV40 polyA fragments were cloned from PX458-mCherry and pCS2 (Addgene, Watertown, MA, USA), respectively. Finally, the cassette consisting of the left arm, T2A mCherry, SV40 polyA, and the right arm fragment in order was inserted to the plasmid backbone. The PCR primers used for cloning all fragments were designed with 20 bp homologous arms for seamless cloning.

The synthesis of the ssDNA donor was performed using an asymmetric primer concentration method [42]. Briefly, PCR was performed with a standard protocol using forward (5′-CAAGGAGTGTTGCCCTGTGT-3′) and reverse (5′-CTCTGGGGTTGACGATAGAG-3′) primers at working concentrations of 250 nM and 16.67 nM, respectively, to generate a mixture of ssDNA and dsDNA. Then, a small portion of the PCR product was digested with Exonuclease I (Takara, Kusatsu, Japan, 2650A) at 37 °C for 30 min and analyzed by gel electrophoresis, and only the ssDNA can be cleaved. After identifying the ssDNA band, the corresponding band from the remaining PCR product was excised from the gel and purified using a standard method.

### 4.2. Embryo Manipulation

*X. tropicalis* were maintained and bred at 25 °C [43], while zebrafish were kept at 28.5 °C with a 14 h:10 h light:dark cycle [1]. *X. tropicalis* embryos were produced by in vitro fertilization, dejellied with 2% cysteine (pH 8.2) in 0.1 × Marc’s modified Ringer’s (MMR; 100 mM NaCl, 20 mM KCl, 1 mM MgSO_4_, 2 mM CaCl_2_, 5 mM Hepes, pH 7.8, 0.1 mM EDTA, pH 8.0), and subsequently washed and cultured in 0.1 × MMR. Zebrafish embryos were raised in Holtfreter’s solution (59 mM NaCl, 0.67 mM KCl, 0.76 mM CaCl_2_ and 2.4 mM NaHCO_3_). *X. tropicalis* embryo microinjection was performed in 6% Ficoll in 0.1 × MMR. For F0 CRISPR mutagenesis, synthetic sgRNAs (*Tyr*-sgRNA: 5′-GGCCCTCAGTTTCCATTCTC-3′, *Gch2*-sgRNA: 5′-TATATGAATGGGGAGGCCAA-3′) were produced by GenScript Co. (Nanjing, China). SgRNA (150 pg/embryo) and Cas9 protein (1 ng/embryo, PNABio, Seattle, WA, USA) with or without ssDNA donor (10 pg/embryo) were injected into embryos to generate knockout or knockin mutants. For PTU treatment, blastula-staged *X*. *tropicalis* and zebrafish embryos were cultured in 0.1 × MMR and Holtfreter’s solution (each containing 0.005% PTU; Aladdin, Shanghai, China, Cat. No. P110661), respectively. For the α-MSH response experiment, isolated tadpole tails were first placed in Steinberg’s balanced salt solution [44] until the pigment cells contracted. Then, the tails were incubated with α-MSH (Solarbio, Beijing, China, CLP0120) (0.6 μM), for 10 min. For the KCl response experiment, isolated tadpole tails were incubated directly in KCl (150 nM) for 5 min. All responses of pigment cells were monitored with the inverted fluorescence microscope (keyence, Shanghai, China, BZ-X800). For all analyses, *X. tropicalis* embryos at stage 42 (tadpoles) and zebrafish embryos at dpf5 were utilized.

### 4.3. Genotyping of Gene-Edited Embryos

Embryos were lysed using the lysis buffer (10 mM Tris-HCl, 75 mM NaCl, 25 mM EDTA, 1% SDS, 0.01% proteinaseK), and the genomic DNA were extracted by the phenol-chloroform method to perform PCR. All the genotyping PCRs were performed using GoTaq^®^ Green Master Mix (Promega, Beijing, China, M7122) according to the manufacturer’s protocol.

For the *Tyr*-locus knockin experiment, primer sequences used in genotyping PCR were as follows (5′Fw: 5′-CATTCCCAGTTTGACTTTGCTG-3′, 5′Rv: 5′-TTGAAGCGCATGAACTCCTTGATG-3′; 3′Fw: 5′-CGCCTACAACGTCAACATCAAGT-3′, 3′Rv: 5′-TTGGAGTTGTAACTGAACAACAGA-3′). Then, the target bands were purified and subjected to TA cloning using the One-Stop Zero TOPO-TA Cloning Kit (Sangon Biotech, Shanghai, China, B522227-0040). Finally, the clones were sequenced and mapped to verify the accuracy of fragment integration.

For the *Gch2*-locus knockout experiment, primer sequences used in genotyping PCR were as follows (Fw: 5′-GGGCTTCTCTGCCAGTGTTC-3′, Rv: 5′-CACCATAGACCGTTTCCTTGT-3′). Then, the PCR products were sequenced and mapped to analyze the editing outcomes. To evaluate the editing efficiency of targeting *Gch2*, sequencing chromatograms from a mixed sample of 10 injected and uninjected tadpoles were analyzed using an online tool (https://www.synthego.com/).

### 4.4. Transmission Electron Microscopy

Transmission electron microscopy was performed according to a protocol modified from the previous study [17]. In brief, the stage 42 tadpoles were double fixed with 2.5% glutaraldehyde (R20510, Shanghai yuanye Bio-Technology Co., Ltd., Shanghai, China) at 4 °C overnight and followed with 1% OsO4 at room temperature for 1 h. After fixation, the sample was first dehydrated with a graded series of ethanol (30%, 50%, 70%, 80%), then with a graded series of acetone (90%, 95%, 100% and 100%). Infiltration was performed by placing the sample into a graded series of mixtures of absolute acetone and the final Spurr resin (1:1 for 1 h, 1:3 for 3 h and pure final Spurr resin for overnight) at room temperature. The sample was embedded and heated at 70 °C for more than 9 h. Finally, the sample was sectioned by LEICA EM UC7 ultratome (Leica, Wetzlar, Germany), and the sections were stained by uranyl acetate and alkaline lead citrate for 10 min, respectively, and imaged using Hitachi Model H-7650 TEM (Hitachi, Tokyo, Japan). OsO4 and Spurr resin were purchased from SPI-CHEM Co. (West Chester, PA, USA).

### 4.5. Spectral Analysis and Fluorescence Imaging

For spectral analysis and fluorescence imaging, the stage 42 tadpoles were anaesthetized with 0.1% MS-222 (Sigma, St. Louis, MO, USA) in 0.1 × MMR and mounted in 0.8% (*w*/*v*) low-melting-point agarose (A8350, Solarbio, Beijing, China) dissolved in 0.1 × MMR. Then, a circle of silicone (H121991, Aladdin, Shanghai, China) grease is applied to raise the cover glass for imaging. More operational details were described by a previous study [45]. Full-spectrum emission spectral analysis under different excitation wavelengths was performed using the λ mode of a two-photon microscope (LSM710 NLO, Zeiss, Oberkochen, Germany). The data was obtained using ZEN software (2009 version), and the graphs were plotted using GraphPad Prism9.5.0. The LED fluorescence microscope ( Keyence, Shanghai, China, BZ-X800) and the laser confocal microscope (FV, 3000) were used for fluorescence imaging. The filter parameters used in conjunction with the LED fluorescence microscope are as follows: GFP/RFP, excitation wavelength: 470 ± 20/525 ± 25 nm, excitation wavelength: 545 ± 20/605 ± 35 nm. For the *X. tropicalis Gch2*-knockout and the zebrafish PTU-treatment assays, embryo samples were imaged using the Z-stack mode on the laser confocal microscope with 0.8 µm thickness of the optical section.

### 4.6. Phylogenetic Tree Analysis

The amino acid sequences of genes analyzed were downloaded from NCBI GenBank database. Multiple sequence alignment was performed using the ClustalW tool (https://www.genome.jp/tools-bin/clustalw, accessed on 20 December 2025) with default parameters (Gap Opening Penalty = 10, Gap Extension Penalty = 0.2) to ensure the accuracy and consistency of the sequence alignment. After sequence alignment, the phylogenetic tree was constructed based on the Neighbor-Joining (NJ) method with 1000 bootstrap replicates using MEGA 11 software. For the tree construction, the p-distance model was utilized to estimate sequence evolutionary distances based on amino acid substitutions; concurrently, the partial deletion mode (site coverage cutoff, 50%) was applied to handle missing sites and gaps in the alignment, thus minimizing the impact of data bias on the tree construction results.

## 5. Conclusions

In this study, we found that tyrosinase-deficiency-induced skin mutant melanophores (Td-MMs) in *X. tropicalis* tadpoles exhibit strong autofluorescence under the GFP filter (Figure 8). Further spectroscopic analysis showed that two types of Td-MMs have similar emission spectral characteristics under blue light (405 nm) and green light (543 nm) excitation: both exhibit strong fluorescence under the GFP filter but no detectable fluorescence under the RFP filter; under near-UV excitation (405 nm), their emission spectra differed: only MMs induced by *Tyr* knockout exhibited autofluorescence under the TagBFP filter. By seamlessly integrating mCherry into the *Tyr* locus via gene knockin technology, we confirmed that red fluorescent protein can effectively avoid the interference of autofluorescence from Td-MMs and is suitable for in vivo fluorescence imaging experiments. Furthermore, our results revealed that the synthesis of fluorescent pigment in Td-MMs relies on the Gch2-mediated pterine biosynthesis, which also plays a key role in this process in leucophores of Arabidopsis killifish and xanthophores of zebrafish.

## Data Availability

The original contributions presented in this study are included in the article. Further inquiries can be directed to the corresponding author.

## References

[1] Kapp F.G., Perlin J.R., Hagedorn E.J., Gansner J.M., Schwarz D.E., O’Connell L.A., Johnson N.S., Amemiya C., Fisher D.E., Wölfle U. (2018). Protection from UV light is an evolutionarily conserved feature of the haematopoietic niche. Nature.

[2] How M.J., Santon M. (2022). Cuttlefish camouflage: Blending in by matching background features. Curr. Biol..

[3] Hashimoto H., Goda M., Futahashi R., Kelsh R.N., Akiyama T. (2021). Pigments, Pigment Cells and Pigment Patterns.

[4] Hamied A., Alnedawy Q., Correia A., Hacker C., Ramsdale M., Hashimoto H., Kudoh T. (2020). Identification and Characterization of Highly Fluorescent Pigment Cells in Embryos of the Arabian Killifish (*Aphanius Dispar*). iScience.

[5] Schartl M., Larue L., Goda M., Bosenberg M.W., Hashimoto H., Kelsh R.N. (2016). What is a vertebrate pigment cell?. Pigment Cell Melanoma Res..

[6] Tian X., Cui Z., Liu S., Zhou J., Cui R. (2021). Melanosome transport and regulation in development and disease. Pharmacol. Ther..

[7] Fujii R. (1993). Cytophysiology of Fish Chromatophores. Int. Rev. Cytol..

[8] Lamoreux M.L., Kelsh R.N., Wakamatsu Y., Ozato K. (2005). Pigment pattern formation in the medaka embryo. Pigment Cell Res..

[9] Kelsh R.N., Harris M.L., Colanesi S., Erickson C.A. (2009). Stripes and belly-spots—A review of pigment cell morphogenesis in vertebrates. Semin. Cell Dev. Biol..

[10] Mayor R., Theveneau E. (2013). The neural crest. Development.

[11] Liang W., Hou C., Zhu Z., Wang P., Wang X., Li Z., Xue J., Ran R. (2025). Cutaneous Pigment Cell Distributions and Skin Structure of *Xenopus*. Pigment Cell Melanoma Res..

[12] Kimura T., Nagao Y., Hashimoto H., Yamamoto-Shiraishi Y.-I., Yamamoto S., Yabe T., Takada S., Kinoshita M., Kuroiwa A., Naruse K. (2014). Leucophores are similar to xanthophores in their specification and differentiation processes in medaka. Proc. Natl. Acad. Sci. USA.

[13] Nagao Y., Suzuki T., Shimizu A., Kimura T., Seki R., Adachi T., Inoue C., Omae Y., Kamei Y., Hara I. (2014). Sox5 Functions as a Fate Switch in Medaka Pigment Cell Development. PLoS Genet..

[14] Lewis V.M., Saunders L.M., Larson T.A., Bain E.J., Sturiale S.L., Gur D., Chowdhury S., Flynn J.D., Allen M.C., Deheyn D.D. (2019). Fate plasticity and reprogramming in genetically distinct populations of *Danio* leucophores. Proc. Natl. Acad. Sci. USA.

[15] Le Guyader S., Jesuthasan S. (2010). Analysis of Xanthophore and Pterinosome Biogenesis in Zebrafish Using Methylene Blue and Pteridine Autofluorescence. Pigment Cell Melanoma Res..

[16] Lister J.A. (2019). Larval but not adult xanthophore pigmentation in zebrafish requires GTP cyclohydrolase 2 (gch2) function. Pigment Cell Melanoma Res..

[17] Fukuzawa T. (2004). Unusual leucophore-like cells specifically appear in the lineage of melanophores in the periodic albino mutant of *Xenopus laevis*. Pigment Cell Res..

[18] Fukuzawa T. (2015). Ferritin H subunit gene is specifically expressed in melanophore precursor-derived white pigment cells in which reflecting platelets are formed from stage II melanosomes in the periodic albino mutant of *Xenopus laevis*. Cell Tissue Res..

[19] Hellsten U., Harland R.M., Gilchrist M.J., Hendrix D., Jurka J., Kapitonov V., Ovcharenko I., Putnam N.H., Shu S., Taher L. (2010). The genome of the western clawed frog *Xenopus tropicalis*. Science.

[20] De Robertis E.M., Gurdon J.B. (2021). A Brief History of *Xenopus* in Biology. Cold Spring Harb. Protoc..

[21] Ran R., Li L., Xu T., Huang J., He H., Chen Y. (2024). Revealing mitf functions and visualizing allografted tumor metastasis in colorless and immunodeficient *Xenopus tropicalis*. Commun. Biol..

[22] Diener J., Sommer L. (2021). Reemergence of neural crest stem cell-like states in melanoma during disease progression and treatment. Stem Cells Transl. Med..

[23] Kaufman C.K., Mosimann C., Fan Z.P., Yang S., Thomas A.J., Ablain J., Tan J.L., Fogley R.D., van Rooijen E., Hagedorn E.J. (2016). A zebrafish melanoma model reveals emergence of neural crest identity during melanoma initiation. Science.

[24] Ran R., Li L., Cheng P., Li H., He H., Chen Y., Hang J., Liang W. (2024). High frequency of melanoma incdkn2b^−/−^/tp53^−/−^
*Xenopus tropicalis*. Theranostics.

[25] Ran R., Li L., Shi Z., Liu G., Jiang H., Fang L., Xu T., Huang J., Chen W., Chen Y. (2022). Disruption oftp53 leads to cutaneous nevus and melanoma formation in *Xenopus tropicalis*. Mol. Oncol..

[26] Ran R., Li L., Chen P., Li S., Wang P., Zhu Z., Wang X., Chen Y., Hang J., Liang W. (2025). Versatile *Xenopus tropicalis* model with targeted integration of human BRAFV600E. Proc. Natl. Acad. Sci. USA.

[27] Aslan Y., Tadjuidje E., Zorn A.M., Cha S.-W. (2017). High-efficiency non-mosaic CRISPR-mediated knock-in and indel mutation in F0 *Xenopus*. Development.

[28] Godden A.M., Antonaci M., Wheeler G.N. (2023). An Efficient CRISPR-Cas9 Method to Knock Out MiRNA Expression in *Xenopus tropicalis*. Methods Mol. Biol..

[29] Suzuki K.-I.T., Sakane Y., Suzuki M., Yamamoto T. (2018). A Simple Knock-In System for *Xenopus* via Microhomology Mediated End Joining Repair. Methods Mol. Biol..

[30] Park D.-S., Yoon M., Kweon J., Jang A.-H., Kim Y., Choi S.-C. (2017). Targeted Base Editing via RNA-Guided Cytidine Deaminases in *Xenopus laevis* Embryos. Mol. Cells.

[31] Nakajima K., Nakajima T., Takase M., Yaoita Y. (2012). Generation of albino *Xenopus tropicalis* using zinc-finger nucleases. Dev. Growth Differ..

[32] Nakayama T., Grainger R.M., Cha S. (2020). Simple embryo injection of long single-stranded donor templates with the CRISPR/Cas9 system leads to homology-directed repair in *Xenopus tropicalis* and *Xenopus laevis*. Genesis.

[33] Shi Z., Wang F., Cui Y., Liu Z., Guo X., Zhang Y., Deng Y., Zhao H., Chen Y. (2015). Heritable CRISPR/Cas9-mediated targeted integration in *Xenopus tropicalis*. FASEB J..

[34] Yasutomi M., Hama T. (1972). Electron microscopic study on the xanthophore differentiation in *Xenopus laevis*, with special reference to their pterinosomes. J. Ultrastruct. Res..

[35] Sharma M. (2023). Selecting the Fluorescent Protein for In Vivo Imaging Experiments. Methods Mol. Biol..

[36] Mao C., Zheng L., Zhou Y., Wu H., Xia J., Liang C., Guo X., Peng W., Zhao H., Cai W. (2018). CRISPR/Cas9-mediated efficient and precise targeted integration of donor DNA harboring double cleavage sites in *Xenopus tropicalis*. FASEB J..

[37] Fukuzawa T. (2020). Periodic albinism of a widely used albino mutant of *Xenopus laevis* caused by deletion of two exons in the Her-mansky-Pudlak syndrome type 4 gene. Genes Cells.

[38] Fukuzawa T., Kikuchi Y. (2018). Unusual light-reflecting pigment cells appear in the *Xenopus neural* tube culture system in the presence of guanosine. Tissue Cell.

[39] Ziegler I. (2010). The pteridine pathway in zebrafish: Regulation and specification during the determination of neural crest cell-fate. Pigment Cell Res..

[40] Braasch I., Schartl M., Volff J.-N. (2007). Evolution of pigment synthesis pathways by gene and genome duplication in fish. BMC Evol. Biol..

[41] Yasutomi M., Hama T. (1976). Electron microscopic demonstration of tyrosinase in pterinosomes of the frog xanthophore, and the origin of pterinosomes. Dev. Growth Differ..

[42] Veneziano R., Shepherd T.R., Ratanalert S., Bellou L., Tao C., Bathe M. (2018). In vitro synthesis of gene-length single-stranded DNA. Sci. Rep..

[43] Shaidani N.-I., McNamara S., Wlizla M., Horb M.E. (2020). Animal Maintenance Systems: *Xenopus tropicalis*. Cold Spring Harb. Protoc..

[44] Jones K.W., Elsdale T.R. (1963). The culture of small aggregates of amphibian embryonic cells in vitro. J. Embryol. Exp. Morphol..

[45] Kieserman E.K., Lee C., Gray R.S., Park T.J., Wallingford J.B. (2010). High-magnification in vivo imaging of *Xenopus embryos* for cell and developmental biology. Cold Spring Harb. Protoc..

